# Bayesian network analysis of multi-compartmentalized immune responses in a murine model of sepsis and direct lung injury

**DOI:** 10.1186/s13104-015-1488-y

**Published:** 2015-09-30

**Authors:** Jean A. Nemzek, Andrew P. Hodges, Yongqun He

**Affiliations:** Unit for Laboratory Animal Medicine, University of Michigan, Ann Arbor, MI 48109 USA; Center for Computational Medicine and Biology, University of Michigan Medical School, Ann Arbor, MI USA; Bioinformatics and Systems Biology, Sanford|Burnham Medical Research Institute, La Jolla, CA USA

**Keywords:** Aspiration, Cytokines, Inflammation, Acute lung injury, Bioinformatics

## Abstract

**Background:**

Inflammatory disease processes involve complex and interrelated systems of mediators. Determining the causal relationships among these mediators becomes more complicated when two, concurrent inflammatory conditions occur. In those cases, the outcome may also be dependent upon the timing, severity and compartmentalization of the insults. Unfortunately, standard methods of experimentation and analysis of data sets may investigate a single scenario without uncovering many potential associations among mediators. However, Bayesian network analysis is able to model linear, nonlinear, combinatorial, and stochastic relationships among variables to explore complex inflammatory disease systems. In these studies, we modeled the development of acute lung injury from an indirect insult (sepsis induced by cecal ligation and puncture) complicated by a direct lung insult (aspiration). To replicate multiple clinical situations, the aspiration injury was delivered at different severities and at different time intervals relative to the septic insult. For each scenario, we measured numerous inflammatory cell types and cytokines in samples from the local compartments (peritoneal and bronchoalveolar lavage fluids) and the systemic compartment (plasma). We then analyzed these data by Bayesian networks and standard methods.

**Results:**

Standard data analysis demonstrated that the lung injury was actually reduced when two insults were involved as compared to one lung injury alone. Bayesian network analysis determined that both the severity of lung insult and presence of sepsis influenced neutrophil recruitment and the amount of injury to the lung. However, the levels of chemoattractant cytokines responsible for neutrophil recruitment were more strongly linked to the timing and severity of the lung insult compared to the presence of sepsis. This suggests that something other than sepsis-driven exacerbation of chemokine levels was influencing the lung injury, contrary to previous theories.

**Conclusions:**

To our knowledge, these studies are the first to use Bayesian networks together with experimental studies to examine the pathogenesis of sepsis-associated lung injury. Compared to standard statistical analysis and inference, these analyses elucidated more intricate relationships among the mediators, immune cells and insult-related variables (timing, compartmentalization and severity) that cause lung injury. Bayesian networks are an effective tool for evaluating complex models of inflammation.

## Background

The pathogenesis of inflammation is linked to numerous cell types and soluble mediators which may have causal or simply incidental significance to a specific disease. Their effects vary depending upon their concentration, compartmentalization, timing, and relative relationships to other mediators [1]. This complexity may explain the lack of significant therapeutic breakthroughs for some devastating inflammatory diseases such as sepsis [2–5]. Although current reductionist approaches have yielded some results, the general failure to translate mediator-based therapies from bench to bedside may be due to the narrow focus of experimental investigations [1, 6]. Ideally, experimental investigations would consider multiple scenarios, many mediators and several body compartments in order to identify the factors relevant to a disease. Unfortunately, many traditional types of analyses may not be able to incorporate the complexity of such a design or provide insight into the actual causal relationships between these factors. It has been suggested that complex systems approaches, involving modeling, simulation, systems biology, chaos theory, and network theory can augment the classical, hypothesis-driven approach that has largely failed to provide critical understanding and treatments for severe illness [7–9]. Therefore, a complex systems approach could prove valuable in the study of disease processes such as the inflammation-associated acute lung injury that complicates sepsis.

Many of the factors that compound the development of acute lung injury are difficult to predict or identify in the clinical situation [10]. In some cases, a septic focus elsewhere in the body may indirectly result in lung inflammation that may progress to actual lung failure. This tendency may be further compounded if the lung is injured again. In fact, the “two hit” theory suggests that an inflammatory insult such as trauma or sepsis may prime the immune system to cause an exaggerated response to a direct lung insult and result in greater injury than the additive effects of the individual insults [11]. Several independent, soluble and cellular mediators have been implicated in this disease process in independent experiments representing a static set of conditions. However, the actual combined effect might also depend upon the timing, severity and compartmentalization of the two insults. This suggests the possibility that the role of various mediators may be altered by a number of extrinsic conditions. The development of effective therapeutic interventions is dependent upon a full understanding of these mediators and, therefore, requires analysis of multiple intrinsic and extrinsic variables simultaneously.

We theorized that a Bayesian network (BN) could be used to find the inferred relationships in complex data sets derived from a model of dual inflammatory injuries to the lung. A BN is a representation of a joint probability distribution over a set of random variables. BNs that most accurately describe a given dataset can be learned automatically by searching through large numbers of network topologies and retaining the most significant top-scoring networks. As probabilistic models, BNs represent probabilistic relationships among variables in a domain. Such probabilistic relationships among variables can be inferred by the application of a BN structural learning algorithm to a relevant dataset [12]. BNs are not necessarily causal since the directionality is typically inferred with additional assumptions and analytics or direct experimental evidences [13–15]. In biology, BNs can identify relationships amongst sets of variables (e.g., genes) in various biological pathways [12, 16–19]. BNs are considered to be ideal for modeling complex systems due to many advantages. BNs are relatively agnostic to the complexity of the relationships predicted. A BN can model linear, nonlinear, combinatorial, stochastic and other types of relationships among variables across multiple levels of biological organizations [20]. Capturing such relationships is difficult with more standard bioinformatics tools such as Pearson correlation, clustering or principal component analysis. Owing to their probabilistic nature, BN algorithms are capable of handling noisy data as found in biological experiments (e.g., DNA microarrays and protein arrays). One key advantage of BNs is that prior knowledge can be easily incorporated during modeling [21]. New variables can be easily added to the BN modeling based on existing data (e.g., experimental conditions and phenotype results). Furthermore, the BN modeling results, represented by directed graph including nodes indicating variables and edges between nodes for statistical associations, can be easily interpreted by humans. Potential targets for therapeutics could then be based not only on lists of up- and down-regulated variables, but also on the interaction networks that relate biological variables.

To produce a multifactorial model of acute lung injury, we studied the effects of an indirect insult from septic peritonitis which was induced by cecal ligation and puncture (CLP) in mice. This was examined in concert with a direct lung injury induced by aspiration. Aspiration of gastric contents is a leading cause of pulmonary complications and acute respiratory distress in trauma and intensive care patients [22]. To date, experimental studies of aspiration have focused on a limited number of mediators found within the lung. The known response to aspiration of stomach acid is characterized by dramatic increases of local pro-inflammatory cytokines (TNF-α, IL-6, chemokines) and recruitment of neutrophils [10, 23] which are key mediators of the progressive inflammatory response [24]. Studies of acid aspiration have identified the CXC chemokine human CXCL8/IL-8 and its rodent counterparts, CXCL2/MIP-2α (macrophage inflammatory protein) and CXCL1/KC (keratinocyte-derived chemokine) or CINC (cytokine-induced CXC chemokine) [23, 25, 26] as important signals for pulmonary neutrophil recruitment. When acidic aspirates contain gastric particulates, the lung injury is increased and the CC chemokine, CCL2/MCP-1 (monocyte chemotactic protein), becomes an important mediator of inflammatory cell aggregation around the foreign material [27]. The inflammatory cells, primarily neutrophils and macrophages, produce enzymes, oxygen radicals and other toxic compounds that injure the lungs. This could progressively lead to lung failure. However, the degree of injury could be altered by many, interrelated factors.

Therefore, we modeled septic lung injury complicated by aspiration. To reproduce clinically relevant scenarios, we induced septic peritonitis by CLP then followed that procedure at different intervals (0, 12, or 48 h) with an aspiration event of various intensities (saline, acidic solution, acid solution with particulate material). Within 6 h of the direct lung injury, near the time aspiration injury peaks, we measured multiple inflammatory and anti-inflammatory cytokines and numerous inflammatory cell types in the body compartments local to the injuries (peritoneum and airways) and in the systemic compartment (plasma). The results were analyzed by standard statistical tests and by Bayesian Networks for interpretation of the complex data sets.

In keeping with the two hit theory, we hypothesized that the combination of insults would result in exuberant production of inflammatory mediators and greater injury in the lung. However, this was not the case because lung inflammation and injury were reduced when two insults were given compared to one. Bayesian analysis showed that both the severity of the lung insult and presence of sepsis influenced neutrophil numbers and lung injury. However, specific chemokine mediators were not strongly linked to the sepsis, suggesting that factors other than exacerbated chemokine production in the lung were involved. Bayesian network analysis led to an alternative theory that the two foci of inflammation compete for neutrophils and decrease the numbers available to create lung injury. Further investigation supported this possibility. The Bayesian network analysis of inflammatory mediators, together with the experimental evidences, provided insight into the causal relationships governing inflammatory responses that were not readily apparent in standard statistical analysis of mediators.

## Results

### Statistical analysis demonstrated the severity of lung injury and the impact of concurrent sepsis

**Fig. 1:**
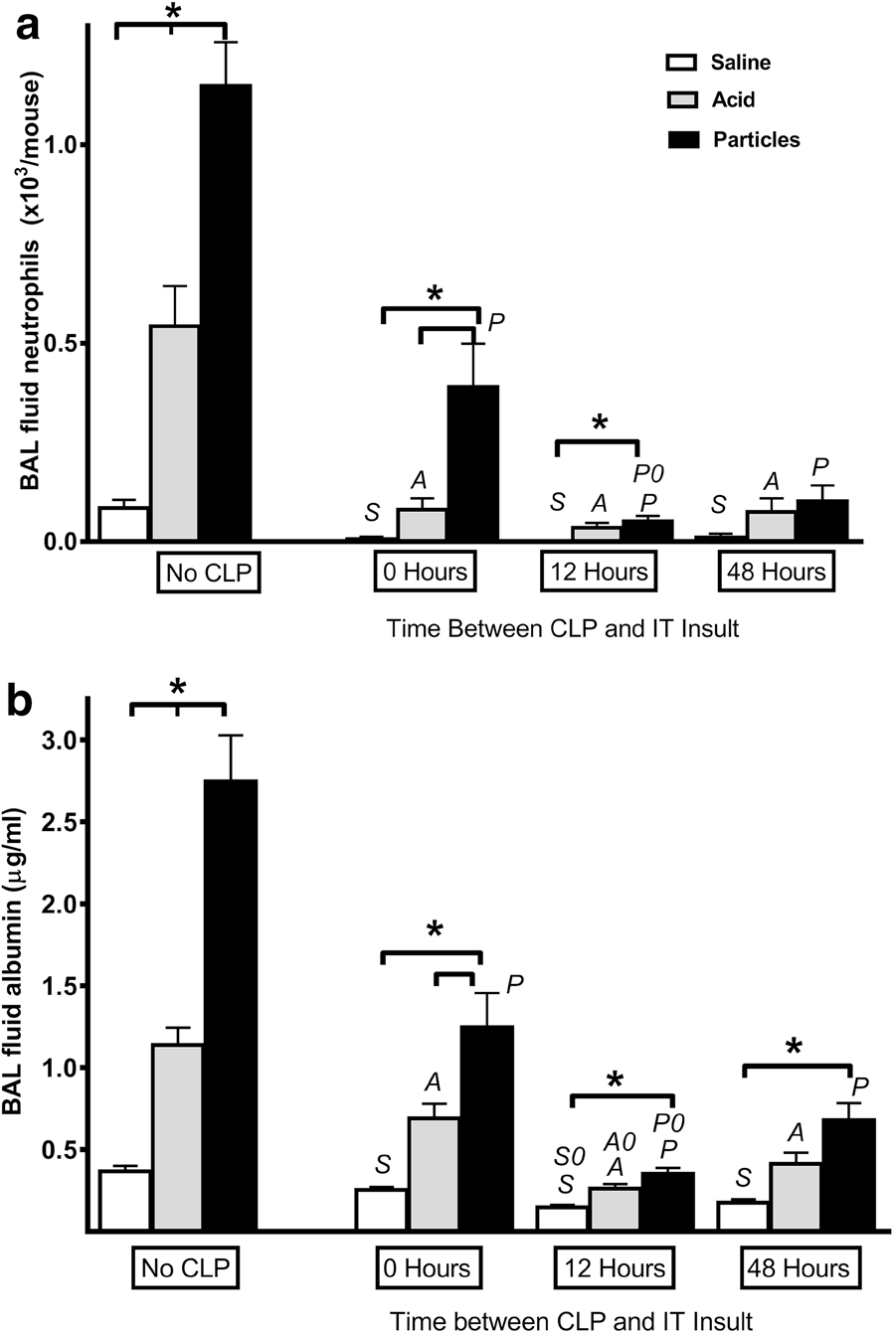
Pulmonary inflammation and injury after aspiration. IT injections of saline, acid, or acid + particles were given to groups of mice (No CLP). In additional groups of mice, cecal ligation and puncture was performed followed by identical IT injections. CLP was performed either immediately before the IT injection (*0* *Hours*) or preceded the IT injections by 12 or 48 h (*12 Hours* or *48 Hours*). All mice were euthanized at 6 h post-IT injection and bronchoalveolar lavage was performed. **a** Neutrophil counts from BAL fluid. **b** Albumin levels in BAL fluid. n = 10–12/group. Results are expressed as mean ± SEM. *p < 0.05, *S* = p < 0.05 compared to No CLP Saline group, *A* = p < 0.05 compared to No CLP Acid group, *P* = p < 0.05 compared to No CLP Particles group; *S0*, *A0*, or *P0* = p < 0.05 as compared to Saline, Acid or Particles group with *0 Hours* interval between insults

To determine the effects of dual insults, lung injury was induced by aspiration of saline, acid or acid + particles in otherwise healthy groups of animals and compared to the lung injury induced by the same aspirates but in animals with concurrent septic peritonitis induced by CLP (0,12, 48 h between insults). Within 6 h of the aspiration insult, samples of bronchoalveolar lavage (BAL) fluid, peritoneal lavage (PL) fluid, and plasma were tested for concentrations of proinflammatory (Table 1) and anti-inflammatory (Table 2) cytokines relevant to the inflammatory injuries. Of particular interest, chemotactic cytokines responsible for the recruitment of cells into sites of inflammation, were also measured (Table 3). The cell counts of numerous leukocytes were also determined (Table 4). Albumin concentration, an indicator of the loss of vascular integrity, was also measured in BAL fluid. From a standard comparison of the means among the groups with lung insult only (No CLP), the data demonstrated progressive and significant increases in the BAL fluid neutrophil counts, as the intensity of the pulmonary insult increased (saline < acid < particles) (Fig. 1a). In addition, the albumin levels demonstrated a similar pattern, indicating a progressive increase in lung injury associated with the increasing inflammation. However, when the aspiration insult was combined with sepsis, the recruitment of neutrophils and accumulation of albumin to the airspaces was actually reduced (Fig. 1a, b). This suppression in animals with CLP was significant (p < 0.05) for each kind of aspirate at all of the time points and contradicted the “two hit” theory. Although timing had an effect at the 12 h insult interval, there was no consistent pattern across the different insult intervals.

**Table 1:** Pro-inflammatory cytokines

Acronym	Cytokine
TNF- α	Tumor necrosis factor-α
IL-6	Interleukin-6
IL-1β	Interleukin-1β
IL-12	Interleukin-12
IFN-γ	Interferon-γ
IL-13	Interleukin-13
IL-4	Interleukin-4
IL-5	Interleukin-5
IL-18	Interleukin-18
IL-2	Interleukin-2

**Table 2:** Anti-inflammatory cytokines in Bayesian network analysis

Acronym	Cytokine
IL-10	Interleukin-10
TNFsr1	Tumor necrosis factor soluble receptor-1
TNFsr2	Tumor necrosis factor soluble receptor-2
IL-1ra	Interleukin-1 receptor antagonist

**Table 3:** Chemokines in Bayesian analysis network

Acronym	Chemokine	Systematic name
MIP-2α	Macrophage inflammatory protein-2α	CXCL2
KC	Keratinocyte-derived chemokine	CXCL1
LIX	Lipopolysaccharide-induced CXC chemokine	CXCL5
MCP-1	Monocyte chemoattractant protein-1	CCL2
MIP-1α	Macrophage inflammatory protein-1α	CCL3
RANTES	Regulated on activation normal T cell expressed and secreted	CCL5
Eotaxin	Eotaxin	CCL11

**Table 4:** Cells in Bayesian analysis network

Acronym	Inflammatory cells
WBC	Total white blood cell
NE	Neutrophil
MO	Monocyte/macrophage
LY	Lymphocyte
EO	Eosinophil

**Fig. 2:**
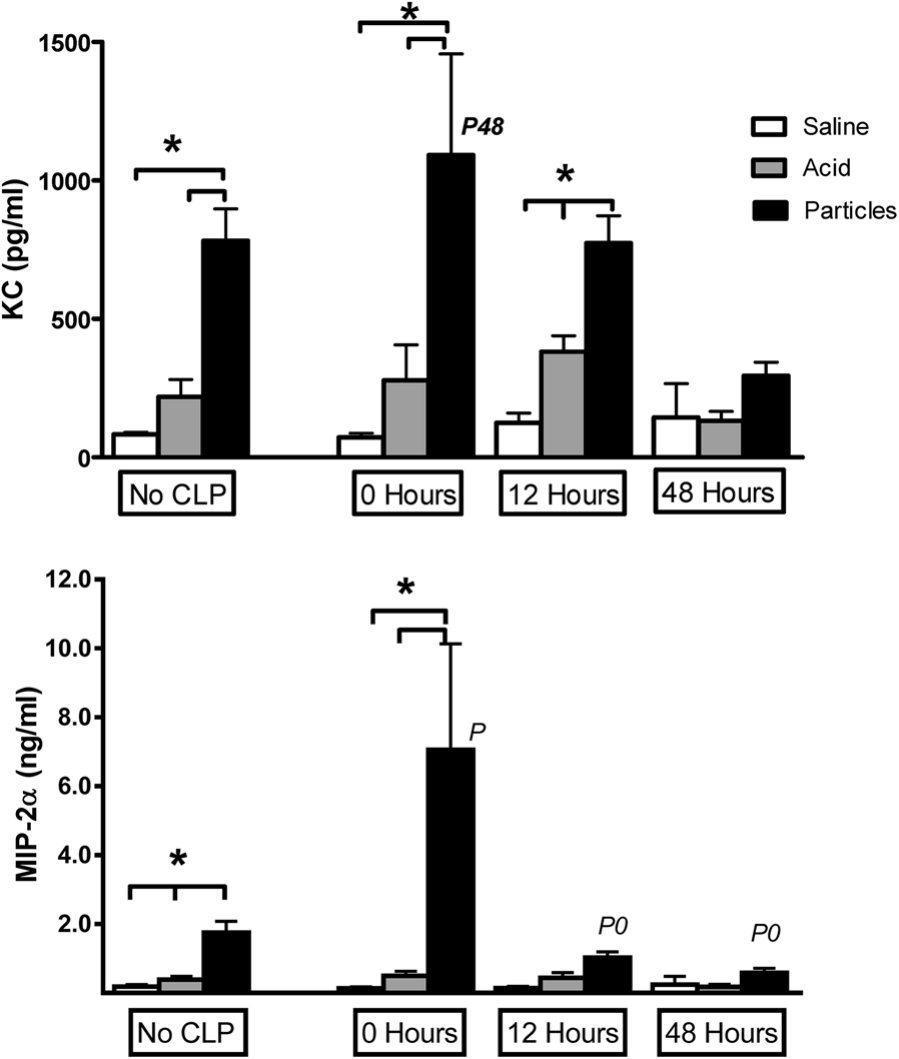
Pulmonary chemokines after aspiration. IT injections of saline, acid, or acid + particles were given to groups of mice (No CLP). In additional groups of mice, cecal ligation and puncture was performed followed by identical IT injections. CLP was performed either immediately before the IT injection (*0 Hours*) or preceded the IT injections by 12 or 48 h (*12 Hours* or *48 Hours*). All mice were euthanized at 6 h post-IT injection and bronchoalveolar lavage was performed. **a** KC concentrations from BAL fluid. **b** MIP-2α concentrations in BAL fluid. n = 10–12/group. Results are expressed as mean ± SEM. *p < 0.05, *P* = p < 0.05 compared to No CLP Particles group, *P0* = p < 0.05 compared to Particles group with 0 Hours interval between insults, *P48* = p < 0.05 compared to Particles group with *48 Hours* between intervals

Previous studies have shown that neutrophil recruitment is a function of chemotaxis induced by CXC chemokines (CXCL1/KC, CXCL2/MIP-2α, and CXCL5/LIX) and also influenced by the CC chemokine, CCL2/MCP-1 [22–24]. The chemokines CXCL1/KC and CXCL2/MIP-2α have been linked to the recruitment of neutrophils after aspiration and their levels correlate with the amount of subsequent lung injury. In this study, the chemokine concentrations found in animals with only aspiration (No CLP) demonstrated a progressive increase as the severity of the aspiration increased (Fig. 2a, b). However, when animals with a specific type of aspiration (No CLP) were compared to animals with the same aspiration paired with sepsis, there were no significant differences in chemokine levels, except for an increase of CXCL2/MIP-2α levels at 0 Hours. This was unusual. Previous literature suggests that the lower neutrophil counts and albumin levels found in animals with aspiration and concurrent sepsis would have been accompanied by lower chemokine levels than animals after aspiration without sepsis. The results were similar for other chemokines such as CXCL5/LIX and CCL2/MCP-1 (data not shown). Our current findings are consistent with previous reports that the type of aspirate definitely affects chemokine concentrations and subsequently the neutrophil counts. However, this offers no explanation for the decrease in neutrophils when aspiration is associated with sepsis or why those neutrophil counts do not trend with the chemokine concentrations when an aspiration insult is paired with sepsis. Consequently, we used Bayesian Networks to examine the same data.

### Bayesian network analysis demonstrated the relative impact of sepsis and aspiration differed between compartments

**Fig. 3:**
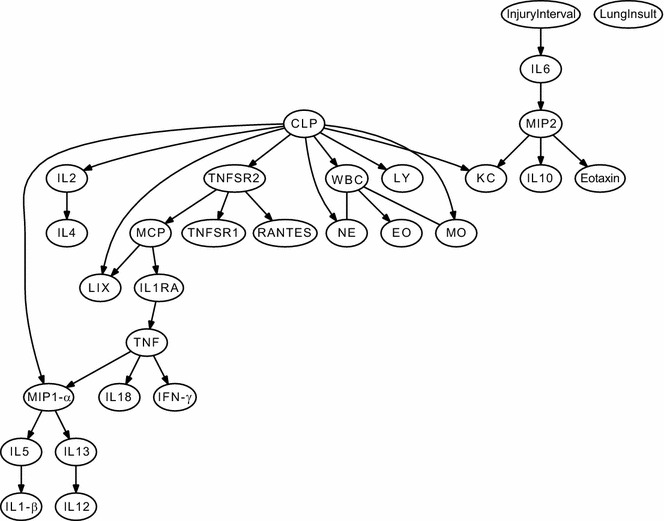
Consensus Bayesian network obtained for peritoneal lavage sample data sets. Mice (n = 10–12/group) were given IT injections of saline, acid, or particles (*lung insult*) with or without the additional insult of cecal ligation and puncture (CLP). CLP was performed at intervals relative to the aspiration injury (*injury interval*), either immediately before the IT injection (0 h) or preceding them by 12 or 48 h. There were a total of 12 combinations of CLP, lung insult and insult interval. All mice were euthanized at 6 h post-IT injection. Peritoneal lavage fluid was collected for cell counts and cytokine levels. The data sets were analyzed in Bayesian Networks. When interactions occurred in the same direction in all of the networks, these are represented as directed edges (*arrows*), whereas those appearing at least once in an opposing direction are represented as undirected edges (*no arrowhead*). CLP refers to the presence of sepsis. Injury interval is the time interval between the induction of sepsis (none, 0, 12, or 48 h) and Lung Insult refers to the aspiration (saline, acid, or particles)

**Fig. 4:**
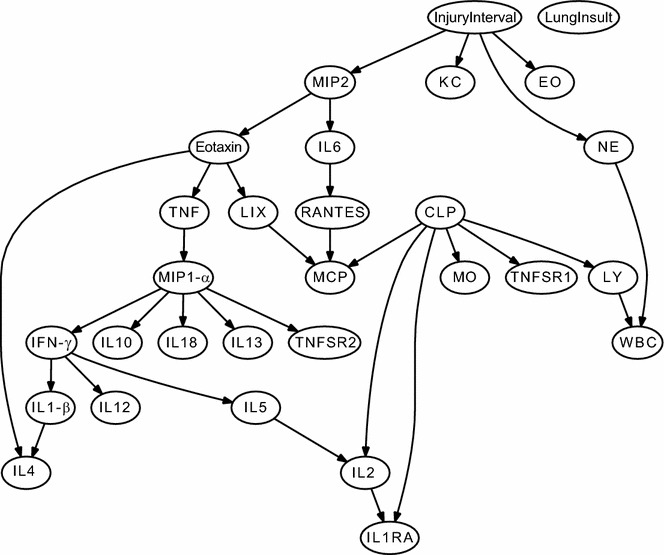
Consensus Bayesian network obtained for blood data sets. Mice (n = 10–12/group) were given IT injections of saline, acid, or particles (*lung insult*) with or without the additional insult of cecal ligation and puncture (CLP). CLP was performed at intervals relative to the aspiration injury (*injury interval*), either immediately before the IT injection (0 h) or preceding them by 12 or 48 h. There were a total of 12 combinations of CLP, lung insult and insult interval. All mice were euthanized at 6 h post-IT injection. Blood was collected and plasma obtained for cell counts and cytokine levels. The data sets were analyzed in Bayesian Networks. When interactions occurred in the same direction in all of the networks, these are represented as directed edges (*arrows*), whereas those appearing at least once in an opposing direction are represented as undirected edges (*no arrowhead*). CLP refers to the presence of sepsis. Injury Interval is the time interval between the induction of sepsis and the lung insult (none, 0, 12, or 48 h) and Lung Insult refers to the aspiration (saline, acid, or particles)

**Fig. 5:**
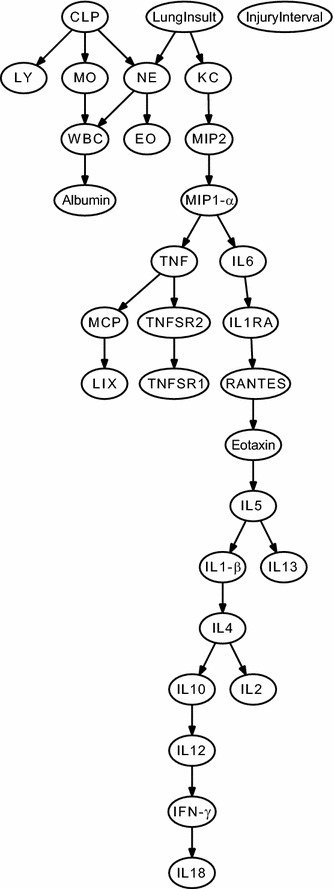
Consensus Bayesian network obtained for BAL fluid data sets. Mice (n = 10–12/group) were given IT injections of saline, acid, or acid + particles (*lung insult*) with or without the additional insult of cecal ligation and puncture (CLP). CLP was performed at intervals relative to the aspiration injury (*injury interval*), either immediately before the IT injection (0 h) or preceding them by 12 or 48 h. There were a total of 12 combinations of CLP, lung insult and insult interval. All mice were euthanized at 6 h post-IT injection. Bronchoalveolar lavage fluid was collected for cell counts and cytokine levels. The data sets were analyzed in Bayesian Networks. When interactions occurred in the same direction in all of the networks, these are represented as directed edges (*arrows*), whereas those appearing at least once in an opposing direction are represented as undirected edges (*no arrowhead*). CLP refers to the presence of sepsis. Injury interval is the time interval between the induction of sepsis and the lung insult (none, 0, 12, or 48 h) and Lung Insult refers to the aspiration (saline, acid, or particles)

The type of lung insult (saline, acid, or particles), the injury interval (0, 12, 48 h) and the presence of sepsis (CLP) were factored directly into the analysis to determine their effects on the mediators. Separate networks were generated for each compartment. Most striking, it was evident that the two disease processes did not directly influence all of the body compartments. For instance, it appeared that the type of Lung Insult was not directly linked to mediators in the distant compartments, peritoneum (Fig. 3) or blood (Fig. 4). However, both the Type of Lung Insult and CLP were directly related to the inflammation in the lung compartment (Fig. 5). This finding was similar to the conclusions eventually drawn from statistical analysis of BAL fluid. However, the Bayesian network analysis recognized this and designated this relationship independent of inferences by an investigator.

### Bayesian network analysis demonstrated variability in relationships between compartmentalized cytokines

The Bayesian networks for individual compartments showed that the relative relationships among cytokine and cell mediators were different in the peritoneal lavage fluid (Fig. 3), blood (Fig. 4), and BAL fluid (Fig. 5). This suggests that the relative importance and interactions of the mediators may differ with location. For instance, the relative relationship between the chemokines KC and MIP-2α changed in direction and proximity among the three sample types. Likewise, the relative association of IL-6 to the chemokines changed with the sample source. These changing relationships suggest that more than one compartment should be analyzed for a true picture of the inflammatory reaction that occurs after more than one insult.

### Pulmonary neutrophil counts were influenced by the septic insult

In the BAL fluid (Fig. 5), the neutrophil counts were directly linked to two parent nodes, the CLP and the Lung Insult. The BN also suggested that both of these factors ultimately influenced the BAL fluid albumin levels, an indicator of vascular leak and amount of lung injury. The directed, fork-like “V” structure formed by the CLP and Lung Insult nodes suggests that the neutrophil activity is co-regulated by more than one factor. Such a “V” structure relationship is typically hard to detect by standard methods and required interpretation of multiple comparisons in our initial analysis. In addition, the “CLP”, “LungInsult”, “NE”, and “EO” form a “Y” structure (Fig. 5), suggesting a causal relation between neutrophil (“NE”) and eosinophil (“EO”) [15]. Therefore, Bayesian network analysis allowed more rapid identification of likely causal relationships. However, it is also noted that the confirmation of the inferred directionality usually needs other evidences, such as the experimental results from the direct perturbation at the upstream lung insult variable.

### Compartmentalized cytokine levels were not always dependent upon the septic insult

In the peritoneal cavity (Fig. 3) and plasma (Fig. 4), the proinflammatory cytokines present (IL-6, TNF-α, and IL-1β) were linked to the CLP and the injury interval. However, the pro-inflammatory cytokines in the BAL fluid (Fig. 5) were connected to the kind of Lung Insult that was given and appeared largely independent of the CLP. Likewise, the chemokine levels were dependent upon the type of Lung Insult and not directly associated with the CLP sepsis (Fig. 5). This was interesting because the neutrophils in the BAL fluid were directly connected to the CLP. Therefore, the BN analysis suggested that factors other than chemokines are important to the relative neutrophil recruitment when aspiration is current with septic peritonitis and that the factor(s) are related to the CLP.

**Fig. 6:**
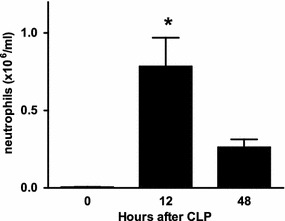
Peritoneal neutrophil counts in response to CLP. Mice had CLP to induce sepsis and were euthanized at 0, 12, and 48 h later. Peritoneal lavage fluid was harvested to reveal neutrophil counts that would correspond to after the time points at which the lung injury was delivered in previous experiments. n-10/group. Results are expressed as mean ± SEM. *p < 0.05 compared to 0 and 48 h groups

According to the BN analysis, the CLP was the primary factor in determining the peritoneal neutrophil count and the localized lung injury did not affect these counts. Based on this finding, we theorized that the lung competes for neutrophils with the peritoneum. To test this, peritoneal lavage fluid was harvested from mice (n = 10/group) at 0, 12, 48 h after CLP. This corresponds to the times after CLP at which the lung injury would have been delivered. The peritoneal neutrophil counts were significantly higher at 12 h than at 0 h (Fig. 6), suggesting a significant number of neutrophils were present at the site of the infection and not available for transport to the lung in spite of high chemokine levels. This neutrophil “sink” would have been stimulated by the CLP, as suggested by the BN, and demonstrates a relative pattern of neutrophil counts that is the opposite of that seen in the BAL fluid of mice when any lung insult was delivered at that time point (Fig. 1).

## Discussion

Since the term “Bayesian networks” was coined by Judea Pearl in 1985 [28] and the BN field formally established in the late 1980s [29, 30], BN has been widely studied at different levels [12, 18, 19, 31]. However, its application in clinical fields is still limited and its real usage in biomedical research has not been vigorously demonstrated. To our knowledge, our study is the first to apply BN analysis to the protein and cellular components of sepsis-associated organ failure. The generally accepted explanation behind the “two hit” theory is that an initial immune response will prime the host for an exaggerated response to a second injury. However, this did not explain the results in our study. Bayesian Network analysis of the data provided further characterization of this complex problem and avenues for further investigation.

In our study, the BN proved useful in the predictive analysis of data derived from a clinically relevant design incorporating multiple scenarios of insult type and timing of the insult. The analysis suggested that the lung inflammation actually had little influence on other body compartments even in the face of concurrent inflammatory disease. However, the neutrophil counts and the pro-inflammatory cytokines in the BAL fluid were affected not only by the kind of Lung Insult but also by the CLP, suggesting the peritoneal response had some influence or communication with the lung. Interestingly, the BN suggested this was not necessarily due to the direct effects of chemokine levels measured near the peak of lung injury. This disconnect could be explained by several possibilities. First, chemokine concentrations in the lung may peak earlier in the course of aspiration lung injury [25, 32, 33], prior to the peak in neutrophil recruitment. Further analysis with a dynamic Bayesian Network Analysis over several time points could demonstrate that relationship in a temporal pattern [9]. Second, the directed chemotaxis of neutrophils is not solely dependent on the absolute chemokine concentration. It may be a function of chemokine gradients between compartments [34]. These relationships were not defined in our analysis and could provide additional answers. Finally, factors other than the induction of chemokine concentrations could explain the influence of the first insult, CLP.

We further examined the possibility that the peritoneum is a neutrophil “sink” after CLP which inhibits neutrophil flux to the secondary lung insult. Previous studies have shown that peritoneal neutrophil counts increase within hours of CLP and remain elevated for several days. Blood neutrophil counts severely decline by 24 h and gradually rebound over several days [25, 35, 36]. Likewise, studies have shown that proinflammatory cytokines in the plasma and peritoneum show distinctive patterns, with significant increases over the first 24 h followed by rapid decline by 48 h [25, 36]. These predictable patterns reinforce the BN analysis. The neutrophil counts in blood and the pro-inflammatory cytokines in blood and peritoneum, (Figs. 1, 2, respectively) were dependent on the timing (insult interval) not just the occurrence of the second insult. Further study in our model demonstrated that the peritoneal neutrophil counts present at the time of the aspiration insult were inversely related to the subsequent BAL fluid counts. Likewise, our studies also showed that the blood neutrophils were significantly lower (p < 0.05) 12 h after CLP (0.8 ± 0.2 × 10^3^ neutrophils/µl) than at the time of CLP (1.9 ± 0.2 × 10^3^ neutrophils/µl). These results suggest the availability of peripheral neutrophils for lung recruitment was finite, regardless of chemokine concentrations. Interestingly, the lack of recruitment to the lung during infection elsewhere may serve as a survival advantage, allowing recruitment of phagocytic cells to the site of infection while protecting the lung from inflammation. Further investigations of the mechanisms behind this advantage could prove useful to protect organs during other inflammatory disease processes.

## Conclusions

In conclusion, the study of inflammatory disease processes involves an extensive group of mediators and experimental factors that cannot be fully appreciated with standard methods of analysis. The examination of single factors or mediators may yield some basic information. For instance, studies of simple aspiration have demonstrated that the production of chemokines in the lung leads to neutrophil recruitment and subsequent lung injury, manifested by increased albumin leakage into airways. A more comprehensive understanding of the disease process may only be derived from consideration of multiple, concurrent factors. In this case, the Bayesian Network analysis provided a tool for early interpretation of the inferred relationships between these factors and helped direct further investigations. Such a BN analysis is generic. Therefore, it can be used to address additional scientific problems in the sepsis field and other research areas. In addition, the procedures and methods used in our experimental analysis can be applied to the practical design of other BNs, stimulating more usage of this valuable tool in biomedical research including clinically oriented fields.

## Methods

### Experimental study design

Mice were randomized into groups. A cecal ligation and puncture (CLP) was performed to induce sepsis in one group, the other had no surgery. Within each group, the mice were randomized to receive an intratracheal instillation of one of three aspirates (lung insult): saline, acid, or acid with particles. In groups with CLP plus Lung Insult, the groups were further randomized for timing of the lung insult relative to the CLP (Injury Interval) at one of three intervals (0, 12, or 48 h). These time points marked the time at which septic inflammation would be none, high, or resolving, respectively. Therefore, there were a total of 12 groups and 10 mice/group. All mice were euthanized at 6 h after the lung insult to allow equal time for development of pathology and evaluation within the time considered the peak of lung inflammation following aspiration. Blood, bronchoalveolar lavage (BAL) fluid, and peritoneal lavage (PL) fluid were obtained from each mouse for multiple cell counts and cytokine analysis.

### Animals

Female ICR mice (23–25 g) were obtained from Harlan Sprague–Dawley, Inc. (Indianapolis, IN). The mice were housed in a temperature-controlled room with a 12-h dark/light cycle. Food and water were given ad libitum. All of the experiments were approved in a protocol (08521) reviewed by the University Animal Care and Use Committee at the University of Michigan.

### Cecal ligation and puncture (CLP)

Mice were anesthetized with isoflurane. The peritoneum was opened and the cecum ligated with silk suture. Two punctures were made with a 26-gauge needle which induces a non-lethal peritonitis. Post-surgery, the mice were given 1.0 mL of warmed saline subcutaneously.

### Aspiration

Mice were anesthetized with isoflurane and given aspirates by the oropharyngeal route as previously described [30]. Aspirates, delivered in two, 40 μl increments per mouse, consisted of one of the following: normal saline (Saline), normal saline titrated to a pH of 1.15 with hydrochloric acid (Acid), or the acidic solution with gastric particles (Particles). Gastric particles were obtained from the stomach contents of healthy mice as previously described [24]. The gastric material was washed with saline, filtered through a 200 µm mesh, autoclaved and resuspended in saline (40 mg/ml). The pH was titrated to 1.15.

### Sample harvest

Mice were anesthetized with 87 µg ketamine (Ketaset; Fort Dodge Laboratories, Inc.) and 13 µg xylazine (Rompun; Bayer Corporation) per gram body weight of mouse. Then, 20 µl of EDTA anti-coagulated blood were obtained from a tail vein for blood counts. Additional whole blood was collected in 50 U of porcine derived heparin (Elkines-Sinn, Inc.). Animals were then euthanized by cervical dislocation. A bronchoalveolar lavage was performed with Hank’s Balanced Salt Solution (without CaCl_2_, Mg_2_SO_4_, and phenol red) by collecting two, one milliliter increments. Peritoneal lavage was performed by injecting 10 ml of HBSS into the abdomen and retrieving 8 ml.

### Peripheral blood analyses

Complete blood counts were performed with a Hemavet Mascot Hematology System Counter 1500R (CDC Technologies, Inc.). The remaining blood samples were centrifuged (2000*g*, 5 min) and plasma was stored at −20 °C for later cytokine analysis.

### Bronchoalveolar lavage (BAL) and peritoneal lavage (PL) fluid cell counts

The BAL and PL fluids were centrifuged (600*g*, 5 min). Supernatants were stored at −20 °C. The pellets from the two samples were pooled, red blood cells lysed with Zap-Oglobin II^®^ (Coulter Corp. Miami, FL, USA), and total cell counts performed with a Coulter Counter model Z1. Slides were loaded with 1 × 10^5^ cells, centrifuged (700*g*, 3 min) and stained with Diff-Quick (Baxter, Detroit, MI, USA). Differentials (300 cells) were counted and used to calculate absolute cell counts.

### Albumin enzyme-linked immunosorbent assay (ELISA)

Standards (mouse albumin, Sigma) and BAL samples were diluted in borate buffer. After overnight incubation at 4 °C, Blocker™ Casein in PBS (Pierce) was used to inhibit non-specific binding. Rabbit polyclonal antibodies against mouse albumin were allowed to incubate for 1 h (6.9 µg/ml in 10 % Blocker™ Casein in PBS) followed by 1 h incubation of goat anti-rabbit IgG conjugated to horse-radish peroxidase (Jackson ImmunoResearch Laboratories; 1:8000). 3, 3′, 5, 5′ tetramethyl benzidine (TMB) was used as the color reagent and 1.5 N sulfuric acid was used to stop the reaction. The absorbance was read at 465 and 590 nm.

### Cytokine microarray

All cytokines except KC were measured with a microarray immunoassay [37]. The microarray quantified cytokines considered to be pro-inflammatory (Table 1) or anti-inflammatory (Table 2). In addition, the microarray included several chemokines (Table 3). Capture antibodies (R and D Systems) were applied to ELISA plates using a non-contact (Piezorray) spotting system. Plates were blocked with Blocking Buffer (LI-COR, Inc.) for 1 h and then washed with buffer (Schleicher and Schuell). Standards and samples were incubated overnight at room temperature with constant shaking. After washing, the matched antibodies, conjugated to biotin, were incubated for 1 h. The plates were washed and streptavidin (IRDye 800, 1.0 mg/ml) was incubated for 30 min in the dark with constant shaking. The plate was read on an Odyssey infrared imaging system. Standard curves were created with Statlia software.

### CXCL1/KC ELISA

A separate KC ELISA was run due to complications of crossreactivity with other cytokines on the microarray. The BAL fluid, PL fluid and plasma were diluted 1:2, 1:2, and 1:10, respectively. Matched antibody pairs and recombinant mKC (R&D Systems) were used in an indirect ELISA [38] with a detection system of biotinylated antibody, peroxidase-conjugated streptavidin (Jackson ImmunoResearch Laboratories) and 1 % TMB. Absorbance was read at 450 and 630 nm.

### Bayesian network analysis

The BANJO BN analysis tool (http://www.cs.duke.edu/~amink/software/banjo/) was used as the backend BN executor [20, 39]. BANJO includes both static and dynamic Bayesian network searching given an underlying dataset, and either null or prior assumptions which can be fixed or flexibly changed. In this study, the BANJO software was used to discretize immunological data prior to simulated annealing searches of a relatively large network space using no structural priors and the Bayesian Dirichlet scoring metric [31, 40]. The BANJO method has been used in different studies [39, 41, 42]. In the current static BN analysis, raw data for each cytokine and cell count measured were discretized or binned into 3 states. The discretization criteria for individual factors were generated manually based on expert evaluations, while no structural priors (or edges) in the network were assumed a priori to prevent any bias or circular reasoning. Then, separate simulations were run on the descretized cytokine and cell count data for each of the three sample sources: (1) Peritoneum, (2) Blood, and (3) BAL. A fourth simulation tested the relations using combined datasets from all three locations to identify common associations among all possible data. In this combinatory network analysis, we generated a variable called “type” to indicate the fluid source for each dataset. Three additional variables were generated. The CLP had two values 0 or 1, representing the implementation of CLP procedure or not. Lung injury had four values (states): 0 for no injury, 1 for saline treatment, 2 for acid treatment, and 3 for treatment with particles. Injury interval also had 4 states (0 HR, 12 HR, or 48 HR representing the time between delivery of CLP and the lung insult and None denoting that no lung insult had been given). Each simulation was run for 50 h in our BN execution. For each of the sample sources, 5 × 10^9^ Bayesian networks were searched (50 simulations with 1 × 10^8^ networks/simulation searched for combined sample sources, 100 replicate simulations with 5 × 10^7^ for the peritoneum, blood, or BAL sample sources). For each analysis, consensus networks were generated using the best Bayesian networks sharing the top log posterior probability. Interactions with arrows appearing in the same direction in all of the networks are represented as directed edges (or arrows), whereas those appearing at least once in an opposing direction are represented as undirected edges (no arrowhead). A direct linkage between two factors in a BN graph indicates that these two factors are more closely associated with high probability support than those factors that do not have direct linkages.

### Statistical analysis

Summary data were expressed as mean ± SEM. Student’s *t* test and ANOVA with post hoc Tukey’s test were used to analyze differences among groups.

## Abbreviations

BN: Bayesian Network
CLP: cecal ligation and puncture
TNF-α: tumor necrosis factor alpha
IL-6: interleukin 6
IL-1β: interluekin beta
CXCL: CXC ligand
MIP-2α: macrophage inflammatory protein
KC: keratinocyte-derived chemokine
CINC: cytokine-induced CXC chemokine
MCP-1: monocyte chemotactic protein
BAL: bronchoalveolar lavage
PL: peritoneal lavage
LIX: lipopolysaccharide-induced CXC chemokine
NE: neutrophil
EO: eosinophil
TMB: 3, 3′, 5, 5′ tetramethyl benzidine
